# Glycerolic Licorice Extracts as Active Cosmeceutical Ingredients: Extraction Optimization, Chemical Characterization, and Biological Activity

**DOI:** 10.3390/antiox8100445

**Published:** 2019-10-01

**Authors:** Petar Ciganović, Katarzyna Jakimiuk, Michał Tomczyk, Marijana Zovko Končić

**Affiliations:** 1 Department of Pharmacognosy, Faculty of Pharmacy and Biochemistry, University of Zagreb, A. Kovačića 1, 10000 Zagreb, Croatia; petar.ciganovic@me.com (P.C.); katarzynajakimiuk1@gmail.com (K.J.); 2 Department of Pharmacognosy, Faculty of Pharmacy, Medical University of Białystok, ul. Mickiewicza 2a, 15-230 Białystok, Poland; michal.tomczyk@umb.edu.pl

**Keywords:** licorice, anti-inflammatory, antioxidant, cosmetic, elastase inhibitory activity, green extraction, tyrosinase inhibitory activity

## Abstract

A green ultrasound-assisted extraction (UAE) method using glycerol/water mixtures for extraction of licorice (*Glycyrrhiza glabra*) bioactive constituents was developed in this study. The response surface method, according to the Box-Behnken design, was employed to optimize the extraction parameters: glycerol concentration (X_1_), temperature (X_2_), and the amount of herbal drug used in the production (X_3_). The responses were content of total phenols (TP), TP extraction efficiency (TPy) and the content of licorice characteristic constituents, glabridin (Gla) and isoliquiritigenin (Iso). Response surface analysis predicted the optimal extraction conditions for maximized amounts of TP, Tpy, Gla, and Iso. The extracts were prepared using the calculated conditions. The analysis of the selected constituents confirmed the validity of the model. Furthermore, biological activity of the extracts was tested. The results demonstrate that UAE using glycerol is a fast and efficient method for preparation of extracts with excellent radical scavenging, Fe^2+^ chelating and antioxidant activity. Furthermore, the observed notable tyrosinase and elastase inhibitory activity of the extracts, as well as their anti-inflammatory activity, indicate the anti-aging properties of the investigated extracts. The fact that the extracts were prepared using the safe, cosmetically active solvent, glycerol, makes them suitable for direct use in specialized cosmeceutical formulations.

## 1. Introduction

The growing importance of physical appearance in the last century has led to an expansion of sophisticated beauty products purported to have high, almost pharmaceutical, efficacy, sensorial advantages, and safety. Such products, popularly called cosmeceuticals, are applied on the human skin, making it appear younger and healthier. Even though the word “cosmeceutical” is a marketing, rather than a legal term, it is often used in lay language because it reflects both the intended dual activity of such products, as well as the consumers’ expectations. Furthermore, the products that are derived from natural sources, such as plants, are in special demand, not only due to the consumers preferences for natural skincare, but also because of their numerous beneficial effects on human skin [1].

Before being incorporated into cosmetic products, the bioactive principles of plants need to be extracted from crude plant material. The selection of an appropriate extraction method is one of the key steps to consider before proceeding to cosmeceutical formulation development. Failure to do so could lead to the loss of active compounds, hence resulting in the loss of biological activity. However, in addition to displaying the desired biological properties, the extracts used in modern cosmetic products have to fulfill other requirements. Besides stability, safety, and sensory properties, new concerns about environmental impact or animal welfare with respect to the cosmetic development, manufacturing, and quality control are constantly emerging, and new products are being developed in order to meet such needs [2]. For example, the design of green and sustainable extraction methods for natural products is currently a hot research topic in the multidisciplinary area of applied chemistry, biology, and technology. Solvents used for extraction should ideally have a high dissolving power, be biodegradable, non-toxic, and non-flammable. Ethanol, due to its biodegradability and natural origin, fulfills some of the requirements for a green solvent, and it is still among the most used solvents for extraction of natural compounds. However, besides being a relatively good solvent for a wide range of natural products, ethanol is highly flammable and has skin-irritant properties. Thus, efforts are being made to replace ethanol with other solvents, preferably of natural origin [3].

One of the solvents that could effectively replace ethanol is glycerol, a natural, non-toxic, biodegradable liquid, manufactured from renewable sources [4]. Due to the hygroscopic nature of glycerol, it is already widely used for formulation of creams and lotions [3]. Therefore, the glycerol extracts of medicinal plants have a dual role in cosmetic products—as humectants and active agents [3]. Furthermore, the use of glycerol in the finished product means that the removal of the solvent from the cosmeceutical extract is redundant. This renders the glycerolic extraction of medicinal plants highly acceptable from an energy-saving point of view. Interestingly, in spite of all the aforementioned favorable characteristics of glycerol extraction, the use of this solvent for extraction of natural products is still under-researched. Relatively few examples include the use of glycerol for extraction of phenolic antioxidants from two *Artemisia* species [5], grapefruit peels [6], *Hypericum perforatum,* and olive (*Olea europaea*) leaves [7]; as well as stevioside from *Stevia rebaudiana* [8].

Licorice (*Glycyrrhiza glabra* L., Fabaceae) is a perennial plant, well-known for its sweet-tasting root. It contains a wide array of bioactive natural products. Glycyrrhizin, the sweet principle of licorice root is a triterpene-type saponin that displays antiviral, anti-inflammatory, antitumor, and antimicrobial properties [9]. Besides glycyrrhizin, phenolic components, such as chalcone isoliquiritigenin and isoflavonoid glabridin are also important for the observed biological activity of licorice root. *G. glabra* has been traditionally used for promotion of wound healing. Licorice root extracts protect the skin against oxidative stress injuries [10,11], accelerate wound epithelization, ameliorate remodeling at the wound site [12], and efficiently reduce the symptoms of atopic dermatitis (AD). Furthermore, isoliquiritigenin was also found to be beneficial for the treatment of AD-like skin lesions in mice, giving hope that it could be a potential therapeutic agent for the treatment of AD in humans [13]. Glabridin has many properties potentially beneficial in cosmeceutical products. It acts as antioxidant, estrogenic, anti-inflammatory, and skin-whitening agent [14]. It displays skin depigmentation activity and is being incorporated in topical products intended specifically for that purpose [15].

*G. glabra* extracts and its constituents display a wide array of activities potentially useful in cosmetic and dermatologic products. The aim of this work was extraction optimization of phenolic compounds from licorice root using glycerol, a non-toxic and eco-friendly solvent. Skin-related biological activities (antioxidant, enzyme inhibiting and anti-inflammatory) of the prepared extracts were investigated with the aim of obtaining highly active extracts suitable for use in cosmeceutical products.

## 2. Materials and Methods

### 2.1. Chemicals

Reagents, standards and enzymes were purchased from Sigma-Aldrich (St. Louis, MO, USA). The purity of the standards was butylated hydroxyanisole (BHA, ≥98.5%), glycyrrhizic acid ammonium salt (≥95.0%), glabridin (Gla) (≥98.0%), and isoliquiritigenin (Iso) (≥98.0%). Methanol and acetonitrile were HPLC grade. Other reagents and chemicals were of analytical grade.

### 2.2. Plant Material

The plant material (licorice root) was donated by the Suban company (Samobor, Croatia). The exact licorice species was determined using HPLC. The material was confirmed to be *G. glabra* based on the presence of Gla [16]. The presence of other related species was excluded by the absence of quercetine (*G. uralensis*) [17] and licochalcone A [16]. The identity was additionally confirmed using a pharmacopoeial monograph [18]. A voucher specimen is deposited in the Department of Pharmacognosy, Faculty of Pharmacy and Biochemistry, University of Zagreb.

### 2.3. Preparation of the Extracts

The root was milled and passed through a sieve of 850 μm mesh size. Powdered plant material of differing weights (0.6–1 g) was suspended in 10 g of the appropriate solvent (10–90% glycerol in water, *w*/*w*) in a 50 mL Erlenmeyer flask. The extraction was performed in an ultrasonic bath (Bandelin SONOREX^®^ Digital 10 P DK 156 BP, Berlin, Germany) at ultrasonication power of 360 W and frequency of 35 Hz during 20 min. The bath was temperature-controlled (20–70 °C). Upon the extraction, the mixtures were filtered. All the extracts were stored at −20 °C, in the dark.

### 2.4. Spectrophotometric Determination of Total Phenol Content

Total phenols (TP) content was determined using the modified Folin–Ciocalteu colorimetric method [19], by mixing 80 μL extract solution, 80 μL of Folin–Ciocalteu reagent and 80 μL of 10% sodium carbonate solution. After 1 h, absorbance at 630 nm was measured (The FLUOstar^®^ Omega, BMG Labtech, Offenburg, Germany and Stat Fax 3200 reader, Awareness Technologies, Palm City, FL, USA). TP was expressed as mg/g of dry weight from calibration curve recorded for gallic acid.

### 2.5. Spectrophotometric Determination of Total Flavonoid Content

Total flavonoid (TF) content was determined using modified Folin–Ciocalteu colorimetric method [20], by mixing 120 μL extract solution and 120 μL of 0.2% AlCl_3_ solution. After 1 h, absorbance at 420 nm was measured. TF was expressed as mg/g of dry weight from calibration curve recorded for quercetin.

### 2.6. RP-HPLC-DAD Determinations of Glycyrrhizin, Glabridin and Isoliquiritigenin

Prior to the analysis, the extracts were filtered through a 0.45 μm PTFE syringe filter. Quantifications were performed using an HPLC instrument (Agilent 1200 series, Agilent Technologies, Santa Clara, CA, USA) equipped with an autosampler and a DAD detector. Injection volume was 10 μL. The peak assignment and identification was based on comparison of UV/VIS spectra and retention times of peaks in sample chromatogram with that of the standards. Quantification was performed using the respective standard calibration curve. The calibration curves, limit of detection (LD), and limit of quantification (LQ), were determined according to [21] (Table 1). For determination of glycyrrhizin, the modified European pharmacopoeia method [18] was used. Separation was performed on a Nucleodur 100-5 C18 column (Macheray-Nagel, Düren, Germany) column. A mixture of glacial acetic acid, acetonitrile, and water (6:30:64 V/V/V) was used as mobile phase. Separation was performed at 25 °C using flow rate of 2 mL/min. Glycyrrhizic acid ammonium salt was used as a standard for construction of calibration curve. The content of Gla and Iso was determined by a modified method described by Tada et al. [22] on the Zorbax Eclipse XDB-C18 (5 µm, 12.5 mm × 4.6 mm, Agilent, Santa Clara, CA, USA). Mobile phase (water:acetonitrile) was used according to the following protocol 0–3 min (7:3), 53-60 min (2:8). Flow rate was 1.0 mL/min. Gla and Iso were used as standards for the construction of calibration curves.

### 2.7. Extraction Optimization

The experiment was planned using Box-Behnken design (BBD) in Design Expert software v. 8.0.6 (Stat-Ease, Minneapolis, MN, USA). The ranges of design parameters (independent variables) were: glycerol concentration (X_1_, 10–90%, *w*/*w*), temperature (X_2_, 20–70°C), and drug weight (X_3_, 0.6–1g) used for the extraction. TP content, TP/X_3_ ratio (TPy), as well as the Gla and Iso content of the extracts were dependent variables. Response-surface methodology was used to find the relationship between dependent and independent variables. Experimental data was analyzed by multiple regression analysis and fitted to the appropriate polynomial models. The validity of the model was confirmed by the analysis of variance (ANOVA). *p* values < 0.1 were considered statistically significant.

### 2.8. Radical Scavenging Activity

Radical scavenging activity (RSA) was evaluated using the stable 2,2-diphenyl-1-picrylhydrazyl (DPPH) free radical [23]. In short, DPPH solution (0.21 mg/mL, 70 μL) was added to the extract solution (130 μL). After 30 min, the absorbance was recorded at 545 nm. DPPH solution with methanol instead of the extract served as the negative control. RSA was calculated according to the following equation:

$$RSA\;\left(\%\right)=\frac{A_{control}-A_{sample}}{A_{control}}\times 100$$

where *A_control_* is the absorbance of the negative control and *A_sample_* is the absorbance of the respective extract. Concentration of the extract, which scavenges 50% of free radicals present in the solution (RSA IC_50_), was calculated. BHA was used as the standard radical scavenger.

### 2.9. Fe^2+^ Chelating Activity

The chelating activity (ChA) of the investigated substances toward ferrous ions was studied, as described in [24]. To the solution of extract in methanol (150 μL), 0.25 mM FeCl_2_ solution (50 μL) was added. After 5 min, 100 μL of 1.0 mM ferrozine solution was applied. Absorbance at 545 nm was recorded after 10 min. Reaction mixture containing methanol (150 μL) instead of extract served as a control. ChA was calculated using the following equation:

$$ChA\;\left(\%\right)=\frac{A_{control}-A_{sample}}{A_{control}}\times 100$$

where *A_control_* is the absorbance of the negative control and *A_sample_* is the absorbance of the respective extract. Concentration of the extract, which chelates 50% of Fe^2+^ present in the solution (ChA IC_50_), was calculated. EDTA was used as the chelating standards.

### 2.10. Antioxidant Activity in β-Carotene-Linoleic Acid Assay

AOA was evaluated using the β-carotene-linoleic acid system according to modified literature procedure [25]. Aliquots (200 μL) of the emulsion containing β-carotene (6.7 μg/mL), linoleic acid (0.7 mg/mL), and Tween 40 (6.7 mg/mL) were added either to methanol (50 μL) (control) or to the solutions of the extract in methanol (50 μL). The reaction mixture was incubated at 50 °C. The antioxidant activity in β-carotene linoleic acid assay (AACL) was calculated based on the absorbances recorded after 60 min using the following equation:

$$AACL\;\left(\%\right)=\frac{A_{sample}}{A_{control}}\times 100$$

where *A_control_* and *A_sample_* are the absorbances of the water control and antioxidant, respectively. Concentration of the extract that protects 50% β-carotene present in the solution (AACL IC_50_) was calculated. BHA was used as the standard antioxidant.

### 2.11. Tyrosinase Inhibitory Activity

Tyrosinase inhibition activity by the extracts was determined following a method described by [19] with some minor modifications. In 80 μL extract solution, 40 μL of tyrosinase solution (in 16 mM pH 6,8 phosphate buffer) was added. The solution was incubated in dark at 25 °C. After 10 min, 80 μL of L-DOPA solution (0.19 mg/mL in phosphate buffer) was added. After an additional 10 min, the absorbance at 492 nm was measured. Negative control contained a buffer instead of the extract solution. Tyrosinase inhibitory activity (TyInh) was calculated as:

$$TyInh\;\left(\%\right)=\frac{A_{control}-A_{sample}}{A_{control}}\times 100$$

where *A_control_* is the absorbance of the negative control and *A_sample_* is the absorbance of the respective extract. Concentration of the extract, which inhibits 50% of tyrosinase activity (TyInh IC_50_), was calculated. Kojic acid was used as the standard inhibitor.

### 2.12. Elastase Inhibitory Activity

To 100 μL of plant extract solution, 1 mM *N*-succinyl-(Ala)_3_-nitroanilide in Tris-HCl buffer (0.1 M, pH 8.0) was added. After 10 min, 25 °C, 25 µl of porcine pancreatic elastase solution was added. The mixture was further incubated at 25 °C for 10 min and absorbance was measured at 410 nm. A reaction mixture containing buffer instead of extract served as the control. Elastase inhibitory activity (ElInh) was calculated as:

$$ElInh\;\left(\%\right)=\frac{A_{control}-A_{sample}}{A_{control}}\times 100$$

where *A_control_* is the absorbance of the negative control and *A_sample_* is the absorbance of the respective extract. Concentration of the extract, which inhibits 50% of elastase activity (ElInh IC_50_), was calculated. Ursolic acid was used as the standard inhibitor [26].

### 2.13. Anti-Inflammatory Activity

Anti-inflammatory activity was evaluated by the heat-induced ovalbumin coagulation method [27] using Perkin Elmer Lambda 25 spectrophotometer (Perkin Elmer, Waltham, MA, USA). The reaction mixture consisted of 0.4 mL of ovalbumin solution, 2.8 mL of phosphate buffered saline (pH 6.4), and 2 mL of the extract solution. The mixtures were incubated at 37 °C for 15 min and then heated at 70 °C for 5 min. After cooling, their absorbance was recorded at 660 nm. The percentage inhibition of ovalbumin denaturation (OvInh) was calculated using the following formula:

$$OvInh\;\left(\%\right)=\frac{A_{control}-A_{sample}}{A_{control}}\times 100$$

where *A_control_* is the absorbance of the negative control and *A_sample_* is the absorbance of the respective extract. Concentration of the extract, which inhibits 50% of the ovalbumin coagulation (OvInh IC_50_), was calculated. Diclofenac sodium was used as the standard inhibitor.

### 2.14. Statistical Analysis

The measurements were performed in triplicate and the results presented as mean ± standard deviation. In order to establish the IC_50_ values, the experiments were performed using different concentrations (4–7 concentrations, depending on the assay). Statistical comparisons were made using one-way ANOVA, followed by Tukey’s post-hoc test for multiple comparisons (GraphPad Prism, San Diego, CA, USA). *p* values < 0.05 were considered statistically significant.

## 3. Results and Discussion

### 3.1. Response Surface Methodology

In this work, efforts were undertaken to optimize the extraction of bioactive phenolics from licorice root. Special attention was given to *G. glabra’s* most prominent phenolic compounds, Gla and Iso. In order to develop a method that is not only efficient but also environmentally friendly, ultrasound-assisted extraction (UAE) was performed using glycerol/water mixtures.

UAE was used as the extraction technique due to its many advantages in comparison with conventional extraction methods, such as maceration and hot reflux extraction. It is characterized by shorter extraction time, reduced organic solvent consumption, and low energy costs [28]. During the UAE procedure, the extraction efficiency was influenced by numerous extraction parameters. These parameters interacted with each other, affecting the extraction efficacy in a more complex way. Therefore, it is important to evaluate the interactions among these parameters. Response surface methodology (RSM) could be adopted to optimize the parameters and obtain the maximum yields of target compounds [29]. In this work, RSM based on BBD was used to optimize the extraction conditions. Selection of solvent greatly influences extraction efficiency due to its physical-chemical properties, such as polarity, viscosity, and volatility. In this work, the proportion of glycerol in water was used as the first independent variable (X_1_). In addition to solvent, temperature, the second independent variable (X_2_), may strongly affect the efficiency of UAE. High temperature may improve the extraction process by reducing the viscosity of the solvent and increasing kinetic energy of the molecules in the solution. However, it may also lead to degradation of sensitive phytochemicals, including phenolic compounds. The influence of weight of the herbal material used for the extraction was investigated as the final independent variable (X_3_). A higher weight of the drug used for the extraction may increase the content of target molecules in the extracts. However, when larger amounts of herbal drugs are extracted with organic/solvent water mixtures, swelling of the herbal material with water may change the proportions of the solvents in the mixture and consequently the polarity of the extraction mixture [30]. In addition, too high drug/solvent ratio may lead to unnecessary waste generation.

The aim of this study was to not only maximize the total extraction yield of the target compounds (TP, Gla and Iso) within the studied extraction parameters range, but also to achieve better utilization of the crude herbal drug. Therefore, maximized TP extraction yield (TPy) calculated as TP/X_3_ was also investigated. The influence of the independent variables on the amount of target substances is presented in Table 2. The results clearly show that the extraction variables have a great impact on the success of the extraction. Depending on the extraction parameters, the amount of TP and Iso change approximately threefold and range from 279.5 μg/mL to 790.6 μg/mL, and 2.00 μg/mL to 5.76 μg/mL, respectively. However, the most dramatic change is observed in the more-than-fourfold increase of the Gla concentration (3.99–17.30 μg/mL). Keeping in mind the skin-related biological activities of Gla, this finding confirms the importance of careful selection of the extraction conditions for cosmeceutical ingredients. Detailed influence of extraction parameters on the selected responses will be presented later.

### 3.2. Fitting the Model

**Figure 1:**
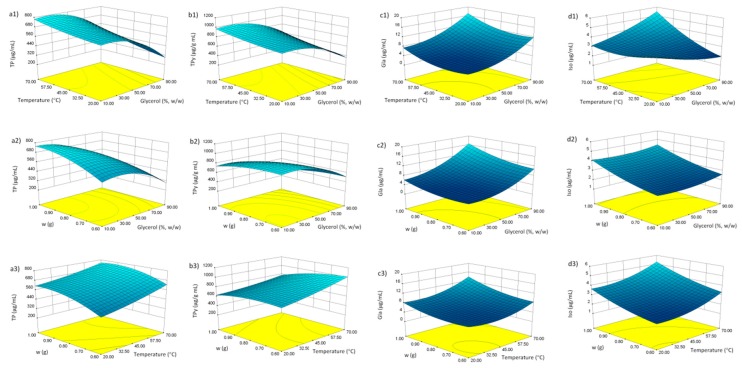
Response surface plots for content of phenols in licorice root extracts: Total phenols (TP) (**a1**–**3**), TP/X_3_ (TPy) (**b1**–**3**), glabridin (Gla) (**c1**–**3**), and isoliquiritigenin (Iso) (**d1**–**3**). For significant model terms, see Table 3.

Multiple regression analysis was used to analyze the experimental results. Table 3 shows the relationship between the independent and dependent variables in the form of polynomial equations. Furthermore, Figure 1 shows the three-dimensional surface and contour plots of the models, which allowed visualizing the effects of the three selected parameters on dependent variables. It can be observed that the glycerol content influenced all the dependent variables as linear term. Furthermore, TP, TPy and Gla were influenced by glycerol content as quadratic term (Table 3). This is clearly visible in Figure 1(a1,2,b1,2), where relatively low glycerol concentration beneficially influenced both TP and TPy extraction efficiency. Gla and Iso concentration, on the other hand, were more favorably influenced by the higher glycerol content (Figure 1(c1,2,d1,2)). This indicates that, unlike the Gla and Iso, the majority of phenols in *G. glabra* root are relatively hydrophilic in nature. Known examples include flavonoid glycosides liquiritin, isoliquiritin, 5,8-dihydroxy-flavone-7-*O*-beta-d-glucuronide, and others [15].

Table 3 shows that the influence of temperature was observed either as linear term (all the dependent variables), quadratic term (Gla), or as the interaction of temperature with glycerol content (Iso). In general, the elevated temperature positively influenced the extraction of phenolics from *G. glabra* root, indicating their good thermostability *G. glabra* (Figure 1(a1,3,b1,3,c1,3,d1,3)). This may be explained by the decreased viscosity of the solvent at high temperature, an effect particularly important in case of viscous solvents such as glycerol.

The positive influence of drug weight as linear term was, expectedly, observed in all the dependent variables (Table 3, Figure 1(a2,3,b2,3,c2,3,d2,3)). In addition, its mild positive influence as quadratic term was observed in Iso extraction. It is interesting to note that a negative, albeit weak, influence of drug weight as quadratic term was observed in case of TP concentration. This may be explained by the property of dry plant material to re-hydrate in a water solution. It may be postulated that the swelling of the material caused the increase in glycerol content, thus changing the composition and polarity of the solvent. This effect is less pronounced with smaller amounts of the herbal drug.

### 3.3. Model Analysis

ANOVA (Table 4) has shown that the relationship between the response variables and independent variables can be satisfactorily expressed using quadratic polynomial equations (Table 3, Figure 1). The statistical significance of each model was calculated using the *F*-test and *p*-values. The calculated *F*-values were higher than 10, while the *p*-values were lower than 0.003. This indicates that the models are highly significant and that they can be used to optimize the extraction variables. Lack-of-fit in the models was statistically insignificant, relative to the pure error which demonstrated that the fitting model is adequate to describe the experimental data. The determination coefficients (*r^2^*) were relatively high (0.9307 ≤ *r*^2^ ≤ 0.9739), showing that the observed values are well replicated by the model. The predicted *r*^2^ were in reasonable agreement with the adjusted ones, further confirming that the models may be used to predict and optimize the amount of target substances in the extracts.

### 3.4. Validation of Optimal Extraction Conditions

Based on the experimental results and statistical analysis, numerical optimizations were conducted in order to establish the optimum levels of independent variables (Table 4). As previously mentioned, the most important extraction factor for majority of the investigated parameters was glycerol concentration. It is well known that the extraction solvent greatly affects extraction efficiency. In this work, the glycerol content needed for optimal extraction of specific phenolic compounds varied according to the response. In general, TP were best extracted using moderate glycerol concentration, as reflected in the maximized TP at 20%. Similar solvent composition was the most suited for extraction of phenolics from grapefruit peels [6]. However, using a slightly higher percentage of ethanol (30% instead of 20%) resulted in better usage of the crude drug, albeit with somewhat lower TP. Gla and Iso, on the other hand, were most efficiently extracted using 85% glycerol. The extraction temperature of 70°C was the best suited for all the desired responses, while the amount of drug needed for the optimal extraction was, expectedly, lowest in the case of TPy. The selected conditions were applied for the preparation of extracts with the desired properties. The predicted results matched well with the experimental ones, with relatively low deviations from calculated values, indicating good suitability of the selected models (Table 5).

### 3.5. Chemical Composition of the Optimized Extracts

In order to test the hypothesis that the optimized extracts are potentially valuable cosmeceutical ingredients, their biological activity was determined using several methods. In addition to that, the prepared extracts were chemically characterized with respect to the main bioactive constituents (Table 6). In accordance with licorice root chemical composition, the prepared extracts were relatively rich in phenolics, especially flavonoids, with notable amounts of their most important representatives, glabridin and isoliquiritigenin. However, the most prominent constituent of the extracts was the saponin glycyrrhizin, the main constituent of licorice root [15]. It was well dissolved in all the applied solvents, and its concentration depended mostly on the weight of the drug used for the extraction (Table 5).

### 3.6. Antioxidant Activity of the Optimized Extracts

Antioxidant activity of the ingredients in cosmetic products is of utmost importance. Firstly, the right antioxidant protects the product against oxidation that occurs during its storage and use [31]. Such influences include free radicals- or metal ions-induced peroxidation of polyunsaturated fatty acids that natural cosmetics are especially rich in. For this reason, the presence of pro-oxidant Fe^2+^ and other ions may, in time, negatively impact not only quality but also safety of the product [32]. Finally, functional cosmeceutical ingredients may have a more active role in such products. They also offer protection against oxidative damage of skin macromolecules associated with the effects of free radicals and UV radiation on the skin [33,34]. Thus, in this work, the influence of the prepared extracts on the free radicals (as modeled by DPPH free radical), chelating activity on Fe^2+^ ions, and the activity in heat-induced unsaturated fatty acid degradation β-carotene-linoleic acid system, were investigated.

**Figure 2:**
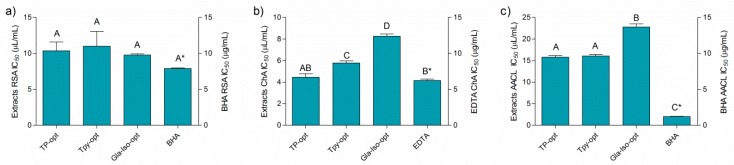
Antiradical (**a**), chelating (**b**), and activity in β-carotene-linoleic acid assay (**c**) and positive controls BHA (butylated hydroxyanisole) and EDTA (ethylenediaminetetraacetic acid). Different uppercase letters indicate statistical significance (*p* < 0.05). Asterisk (*) indicates that the IC_50_ unit is placed on the right *y*-axis.

Figure 2 depicts the results of the antioxidant assays performed in this work. Even though the activity of the extracts may not be directly compared to the standard antioxidants due to the fact that the activity is expressed in different measurements units (the activity of the extracts and standards was expressed as μL/mL and μg/mL, respectively), it is interesting to note that the activity of the extracts and the standards solutions was rather similarly pronounced in all the assays, except for the β-carotene-linoleic acid assay, where BHA was a notably stronger antioxidant (Figure 2a–c). The activity of the individual extracts differed according to the assay. The prepared optimized extracts were similarly efficient radical scavengers with IC_50_ values of approximately 10 μL of extract per mL of solution. In addition, the extracts were able to efficiently chelate Fe^2+^ ions. Among the extracts, TP-opt was the most active ion chelator, followed by TPy-opt. Finally, the extracts inhibited thermally induced degradation of the β-carotene-linoleic acid system (Figure 2). TP-opt and TPy-opt also displayed the strongest, and statistically equal, AACL activity. Comparable activity of TP-opt and TPy-opt indicates that similar effects may be obtained with about 25% less crude drug, which is a finding important from both economical and ecological points of view. In order to test if the solvent contributed to the observed antioxidant activity, the solutions of glycerol, diluted in the same concentrations as it was present in the solutions of the TP-opt, TPy-opt and Gla-Iso-opt at their EC_50_, was tested. However, glycerol displayed no measurable activity in any of the applied antioxidant assays.

### 3.7. Enzyme Inhibiting and Anti-inflammatory Activity of the Optimized Extracts

The activity of the plant extracts in cosmetic products extends beyond simple hydration and antioxidant protection. Therefore, in this work, tyrosinase and elastase inhibitory activity, as well as anti-inflammatory activity against protein coagulation, were investigated. Melanin is a macromolecular pigment that has a photoprotective function in human skin. However, the accumulation of an abnormal amount of melanin in specific skin parts results in hyperpigmented areas and represents an esthetic problem for the affected individual. Tyrosinase is the enzyme responsible for the first step of melanogenesis by catalyzing tyrosine oxidation to dopaquinone. The remainder of the reaction sequence proceeds spontaneously at a physiological pH value. Therefore, tyrosinase inhibitors block melanogenesis and prevent hyperpigmentation of the skin [35]. Specific plant metabolite may protect the skin macromolecules against enzymatic degradation. For example, skin aging and inflammation induced by exposure to UV radiation or other environmental stressors are related to the reduction of production of skin proteins and increased levels of elastase enzymes, which are responsible for elastin breakdown [36]. This damage results in distinctive degenerative changes of the upper dermal connective tissue [37]. Clinical trials confirm that the inhibition of elastase activity indicates the important anti-aging potential of the natural product and other compounds that display it [38]. Skin inflammation can be defined as a skin response to injury, infection, or destruction, normally characterized by heat, redness, pain, swelling or disturbed skin physiological functions [36]. One of the characteristic and causes of inflammatory processes is the denaturation of tissue proteins. Therefore, the suppression of protein denaturation hinders the development of inflammation-related skin changes, which is another important aspect of anti-aging activity [27].

**Figure 3:**
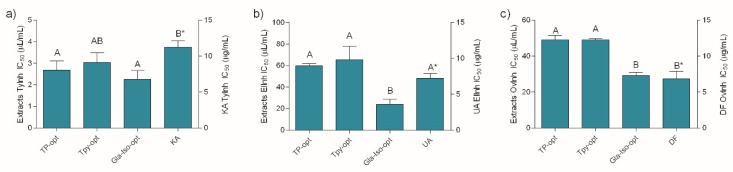
Tyrosinase (**a**) and elastase (**b**) inhibitory, and anti-inflammatory (**c**) activity of the extracts and positive controls KA (kojic acid), UA (ursolic acid) and DF (diclofenac). Different uppercase letters indicate statistical significance (*p* < 0.05). Asterisk (*) indicates that the IC_50_ unit is placed on the right *y*-axis.

As presented in Figure 3, the investigated extracts were excellent tyrosinase and elastase inhibitors, as well as anti-inflammatory agents. Similar to previously described antioxidant assays, the extracts displayed a notable activity in all the assays relative to the positive controls. Keeping in mind the well-established anti-tyrosinase activity of glabridin, the excellent activity of Gla-Iso-opt in this assay was not surprising. However, the other extracts displayed statistically equal activity in this assay. The anti-elastase activity of the Gla-Iso-opt extract, however, was much better pronounced and statistically higher than the activity of the other extracts. Although all the investigated extracts were able to inhibit heat-induced ovalbumin coagulation, the best anti-inflammatory activity was displayed by the Gla-Iso-opt. It is interesting to note that, in accordance with previous findings [39], glycerol itself has a role of an active solvent that prevents the denaturation of proteins such as collagen. Therefore, the influence of glycerol on the heat-induced protein denaturation was also investigated. In order to estimate the proportion of the glycerol activity in the overall activity of the extracts, glycerol was diluted to the same concentration as present in the solutions of the respective extract at its EC_50_. The activity of glycerol, when tested in concentrations present in TP-opt, TPy-opt and Gla-Iso-opt extracts at their EC_50_, was 4.69%, 8.53% and 23.07%, respectively. This means that, at the respective extract’s EC_50_ (e.g., when the activity of the extracts was 50%), glycerol used for preparation of TP-opt only marginally influenced the assay outcome (less than 10%), while glycerol in TPy provided approximately 20% of protection against protein denaturation. However, glycerol presence in the Gla-Iso-Opt extract, which was prepared using 85% glycerol, accounted for 46% of the observed stabilization, while the rest of the activity could be contributed to the specific compounds in the extract and/or their interaction with glycerol. Even though glycerol, when tested at concentrations present at EC_50_ of the extracts in the respective assays did not demonstrate any measurable elastase- or tyrosinase-inhibitory activity, its ability to hinder protein denaturation furthers confirms that the benefits of glycerol extraction for cosmeceutical production extend beyond its application as a green extraction solvent.

## 4. Conclusions

Licorice root contains numerous bioactive natural products, many of which are potent cosmeceutical ingredients. In this work, the UAE method for preparation of licorice root bioactive extracts was optimized. The extraction was performed using mixtures of water with glycerol, a biodegradable, safe, cosmetically active solvent. The prepared extracts displayed excellent radical scavenging, Fe^2+^ chelating, and antioxidant activity. In addition, tyrosinase and elastase inhibitory activity of the extracts, as well as their anti-inflammatory activity, indicated excellent anti-aging properties. Such attractive array of skin-related biological activities makes glycerolic licorice extracts promising constituents of specialized cosmeceutical formulations.

## Figures and Tables

**Table 1:** Slope, intercept and coefficient of determination (*r*^2^) of the calibration curves *, limits of detection (LD) and quantification (LQ) for glycyrrhizin, glabridin, and isoliquiritigenin.

Analyte	Slope (a)	Intercept (b)	*r* ^2^	LD (μg)	LQ (μg)
Glycyrrhizin	257.96	1.54	0.99998638	0.006112	0.018522
Gla	3402.71	26.12	0.9999998	0.000741	0.002246
Iso	5079.81	21.21	0.9999931	0.005013	0.015191

* calibration curves are represented as *y* = a*x* + b, where *y* is the absorbance at the selected wavelength, and *x* is the weight of the analyte (μg).

**Table 2:** Independent variables, their levels for the Box–Behnken design, and the responses obtained.

Run	X_1_	X_2_	X_3_	TP	TPy	Gla	Iso
	(%. *w*/*w*)	(°C)	(g)	(μg/mL)	(μg/g mL)	(μg/mL)	(μg/mL)
1	50	70	0.6	605.3	1008.8	9.12	3.20
2	10	45	0.6	529.6	882.7	4.37	2.48
3	50	70	1.0	753.9	753.9	14.11	5.20
4	50	45	0.8	606.3	757.8	5.29	3.31
5	10	70	0.8	790.6	988.3	6.56	3.11
6	50	45	0.8	779.1	973.9	6.56	2.86
7	50	45	0.8	676.8	846.0	6.09	2.79
8	90	20	0.8	279.5	349.3	12.9	2.00
9	10	45	1.0	748.7	748.7	6.96	4.27
10	10	20	0.8	633.6	792.0	3.99	3.47
11	50	45	0.8	582.2	727.7	4.40	2.07
12	90	70	0.8	518.7	648.3	17.30	5.76
13	90	45	0.6	302.1	503.4	10.14	2.47
14	50	45	0.8	620.8	776.0	6.39	3.29
15	50	20	1.0	638.3	638.3	7.63	3.79
16	90	45	1.0	346.6	346.6	16.18	4.26
17	50	20	0.6	447.4	745.7	5.48	2.02

Independent variables: X_1_ = glycerol content, X_2_ = temperature, X_3_ = weight of the plant material in 10 mL of solvent. TP, TPy, Gla, Iso: concentration of total phenols, TP/X_3_ ratio, glabridin and isoliquiritigenin, respectively.

**Table 3:** Polynomial equations of the models in terms of coded factors.

Response	Unit	The Equation Coefficients: *a* × X_1_^2^ + *b* × X_2_^2^ + *c* × X_3_^2^ + *d* × X_1_ × X_2_ + *e* × X_1_ × X_3_ + *f* × X_2_ × X_3_ + *g* × X_1_ + *h* × X_2_ + *i* × X_3_ + *j*									
		*a*	*b*	*c*	*D*	*e*	*f*	*g*	*h*	*i*	*j*
TP	mg/mL	−113.482 ^a^	16.020	−57.818 ^b^	20.547	−43.631	−10.56	−156.966 ^a^	83.702 ^a^	75.393 ^a^	653.045
TPy	mg/g mL	−144.084 ^a^	22.256	−51.862	25.684	−5.703	−36.864	−195.495 ^a^	109.235 ^a^	−81.642 ^a^	816.306
Gla	μg/mL	2.384 ^a^	2.058 ^a^	1.283 ^a^	0.459	0.863	0.708	4.329 ^a^	2.135 ^a^	1.972 ^a^	5.746
Iso	μg/mL	0.271	0.451	0.237 ^b^	1.032 ^a^	−0.003	0.055	0.146	0.748 ^a^	0.919 ^a^	2.864

X_1_ = glycerol content, X_2_ = temperature, X_3_ = weight of the plant material in 10 mL of solvent. TP, Gla, Iso: concentration of total phenols, glabridin and isoliquiritigenin, respectively. ^a,b^ = The significant equation terms ^a^ = *p* < 0.05, ^b^ = *p* < 0.1.

**Table 4:** Analysis of variance (ANOVA) for the fitted quadratic models for optimization of *G. glabra* extraction process.

	**TP**					**TPy**				
*r* ^2^	*r*^2^ = 0.9329; *r*_A_^2^ = 0.8467; *r*_P_^2^ = 0.8027					*r*^2^ = 0.9325; *r*_A_^2^ = 0.8457; *r*_P_^2^ = 0.8389				
Source	SS	df	MS	*F* Value	*p*-value	SS	df	MS	*F* Value	*p*-value
Model	379,961.7	9	42,218	10.82	0.0024	565,610.6	9	62,845.62	10.74213	0.0025
Lack of Fit	2607.6	3	869	0.14	0.9305	2337.216	3	779.072	0.0807	0.9671
Pure Error	24,713.9	4	6178			38,615.51	4	9653.877		
	**Gla**					**Iso**				
*r* ^2^	*r*^2^ = 0.9739; *r*_A_^2^ = 0.9403; *r*_P_^2^ = 0.7444					*r*^2^ = 0.9307; *r*_A_^2^ = 0.8415; *r*_P_^2^ = 0.6810				
Source	SS	df	MS	*F* Value	*p*-value	SS	df	MS	*F* Value	*p*-value
Model	277.3	9	30.811	29.01	<0.0001	17.23	9	1.915	10.44	0.0027
Lack of Fit	4.24	3	1.412	1.76	0.2926	0.27	3	0.09	0.36	0.7892
Pure Error	3.2	4	0.8			1.01	4	0.253		

SS = Sum of Squares; df = degrees of freedom; MS = Mean Square. *r*_A_^2^ = adjusted *r*^2^; *r*_P_^2^ = predicted *r*^2^. TP, TPy, Gla, Iso: concentration of total phenols, TP/X_3_ ratio, glabridin and isoliquiritigenin, respectively.

**Table 5:** Predicted and observed values for the optimized extracts.

Extract	Measured Response	X_1_	X_2_	X_3_	Resp_pred_	Resp_ms_	RD (%)
		(%, *w*/*w*)	(°C)	(g)			
TP-opt	TP (μg/mL)	20	70	0.93	830.2	854.6	2.9
Tpy-opt	TP (μg/mL)	30	70	0.7	734.8	791.6	7.7
Gla-Iso-opt	Glabridin (μg/mL)	85	70	1	20.67	21.89	5.9
Gla-Iso-opt	Isoliquiritigenin (μg/mL)	85	70	1	6.51	6.23	−4.3

X_1_ = glycerol content, X_2_ = temperature, X_3_ = weight of the plant material in 10 mL of solvent. Resp_pred/ms_ − Predicted and measured response, respectively (units are as in the Measured response column). RD = Response deviation, calculated as (Resp_ms_ − Resp_pred_)/Resp_pred_ × 100.

**Table 6:** Chemical composition of the optimized extracts.

Extract	TP	TF	Gla	Iso	Glycyrrhizin
	(μg/mL)	(μg/mL)	(μg/mL)	(μg/mL)	(mg/mL)
TP-opt	854.6 ± 42.7	667.5 ± 42.7	9.62 ± 0.72	4.02 ± 0.26	4.31 ± 0.22
Tpy-opt	791.6± 48.0	521.4 ± 8.9	8.38 ± 0.17	3.51 ± 0.18	4.20 ± 0.17
Gla-Iso-opt	535.4 ± 32.1	692 ± 32.4	21.89 ± 1.09	6.23 ± 0.16	4.67 ± 0.34

TP, TF, Gla, Iso: concentration of total phenols, total flavonoids, glabridin and isoliquiritigenin, respectively.
